# Comprehensive genomic and metabolomic profiling of *Weissella confusa* UTNCys2-2 highlights bioactive potential

**DOI:** 10.3389/fmicb.2026.1779198

**Published:** 2026-02-27

**Authors:** Gabriela N. Tenea

**Affiliations:** Biofood and Nutraceutics Research and Development Group, Faculty of Engineering in Agricultural and Environmental Sciences, Universidad Técnica del Norte, Ibarra, Ecuador

**Keywords:** CAZyme, exopolysaccharide, metabolic pathways, pangenome, probiotic, *Weissella confusa*

## Abstract

**Introduction:**

The genus *Weissella* comprises a diverse group of lactic acid bacteria (LAB) widely distributed across plant- and food-associated ecosystems and recognized for their functional and technological versatility. *Weissella confusa* UTNCys2-2, a plant-derived strain isolated from Amazonian spiral ginger (*Costus* sp.), that produces exopolysaccharides (EPS) with documented antioxidant activity and promising probiotic properties.

**Methods:**

Whole-genome sequencing of UTNCys2-2 was performed to establish its taxonomic assignment, phylogenomic analysis, while genome mining was conducted to evaluate safety, metabolic potential, and biosynthetic capabilities. Carbohydrate-active enzymes (CAZymes), Kyoto Encyclopedia of Genes and Genomes (KEGG), and MetaCyc pathways were analyzed for functional insights. Moreover, the metabolite composition of the cell-free supernatant (CFS) was examined using liquid chromatography–tandem mass spectrometry (LC–MS/MS) combined with Sequential Windowed Acquisition of all Theoretical Fragment Ion Mass Spectra (SWATH-MS).

**Results:**

The genome consists of a 2.32 Mb circular chromosome (44.59% GC) encoding 2,194 proteins, 76 tRNAs, and 10 rRNAs, with no plasmids. Phylogenomic analyses assigned the strain to the *W. confusa* clade, clustering closely with the reference strain DSM 20196. UTNCys2-2 harbors a complete Type II-A CRISPR-Cas system, intact prophages, and mobile elements, while lacking virulence determinants and transferable antimicrobial resistance genes. Functional annotation revealed 118 CAZymes supporting EPS biosynthesis, polysaccharide utilization, and carbohydrate metabolism. KEGG and MetaCyc pathways highlighted glycogen and riboflavin biosynthesis, stress tolerance, and metabolic versatility. Genome mining identified a Type III polyketide synthase (T3PKS) gene cluster with low similarity to known pathways, suggesting potential for novel secondary metabolites. Pangenome analysis showed extensive strain-specific genes linked to carbohydrate metabolism and EPS production. Metabolomic profiling of the CFS detected alkaloids, bioactive peptides, functional carbohydrates, and phenolics, supporting antimicrobial, probiotic, and host-interactive activities.

**Conclusion:**

*W. confusa* UTNCys2-2 represents a biosafe and metabolically versatile strain with strong genomic capacity for EPS production, potential for novel secondary metabolite biosynthesis, and diverse bioactive properties, supporting its applicability in food fermentation, probiotic development, and microbial biotechnology.

## 1 Introduction

*Weissella* is a diverse genus of LAB known for its broad ecological distribution and functional versatility (Yuan et al., 2021). Species and strains within this genus, including *Weissella cibaria*, *Weissella confusa*, and *Weissella paramesenteroides*, have been isolated from a wide range of environments such as fermented foods (e.g., yogurt, sourdough, kimchi), dairy products, vegetables, soil, human breast milk, and the gastrointestinal tracts of humans and animals (Mun and Chang, 2020; Wang et al., 2020; Yuan et al., 2021). These bacteria play essential roles in fermentation processes, contributing to the texture, flavor, and preservation of foods, and have demonstrated probiotic, biotechnological, and bacteriocinogenic potential (Xia et al., 2019; Pabari et al., 2020). Several strains, particularly *W. confusa*, have been recognized for their ability to inhibit pathogenic bacteria and fungi, support intestinal epithelial regeneration, modulate gut permeability, influence host metabolism, reduce depressive-like behaviors, and even suppress cancer cell proliferation (Dey and Kang, 2020; Sandes et al., 2020; Quattrini et al., 2020; Prado et al., 2020). *W. confusa* has been identified as a promising candidate for direct-fed microbial applications and is listed in the International Dairy Federation Inventory for use in food fermentation (Yuan et al., 2021). Despite this growing body of evidence supporting their health-promoting and industrial potential, further genomic characterization is needed to deepen our understanding of the metabolic pathways, safety, and mechanisms underlying the diverse functionalities of the *Weissella* genus.

Considering the growing interest in the functional diversity and probiotic potential of *Weissella* species, particularly those isolated from fermented foods and animal hosts, recent research has extended this exploration to less-studied ecological niches. In previous investigations, attention was directed toward the microbiota of various Amazonian fruits and flowers, with the aim of identifying LAB strains exhibiting antimicrobial and probiotic properties (Benavides et al., 2016). The ecological origin of these LAB strains plays a pivotal role in shaping their genomic features and functional capabilities, as microbial communities adapt to their environments through evolutionary processes such as horizontal gene transfer (HGT), metabolic specialization, and modulation of stress responses (Tenea, 2022). These findings underscore the importance of environmental context in understanding the functional potential of LAB, including emerging genera like *Weissella*, and support the continued search for novel strains from diverse habitats with applications in health and industry. These microorganisms represent part of the indigenous microflora of their native ecosystems and may serve as promising alternatives to conventional gut-associated bacteria. Their adaptation to unique environmental pressures often endows them with distinctive functional traits, making them attractive candidates for probiotic and antimicrobial applications. Among the various lactobacilli strains evaluated in prior research, only a limited number, particularly coccoid forms, demonstrated notable antimicrobial activity (Tenea and Lara, 2019). However, the antimicrobial potential of peptide-protein extracts derived from UTNCys2-2 was effective against a range of foodborne pathogens (Tenea and Lara, 2019). These findings highlight LAB from underexplored tropical environments as promising sources of bioactive compounds for food and health-related applications. Recent studies have demonstrated the EPS production and *in vitro* probiotic potential of UTNCys2-2, with isolated EPS exhibiting antioxidant activity and distinctive structural features as determined by proton nuclear magnetic resonance (^1^H-NMR), attenuated total reflectance–Fourier transform infrared (ATR-FTIR) spectroscopy, and cytotoxicity assays (Ascanta et al., 2025). However, the present study integrates genome-based analyses and metabolomic profiling to elucidate the taxonomic placement, genomic safety, metabolic potential, and bioactive compound production of UTNCys2-2 relevant to functional food development.

## 2 Materials and methods

### 2.1 Strain information and sequencing workflow

*W. confusa* UTNCys2-2 (GenBank accession no. KY041684.1) was previously isolated from *Costus* sp. (spiral ginger), a wildflower of Cuyabeno (semi-permanently inundated forests flooded by black-water river of Ecuadorian Amazon) (Tenea and Lara, 2019) and routinely grown in MRS broth (Difco, United States). *De novo* whole-genome sequencing and assembly were conducted using the Illumina HiSeq X Ten platform (Macrogen Inc., Seoul, Korea). In brief, the genomic DNA was randomly fragmented, and sequencing adapters were ligated to both 5′ and 3′ ends to construct the sequencing library. The quality of the raw sequencing reads was initially assessed using FastQC v0.11.5, and adapter trimming was performed with Trimmomatic v0.36. Filtered reads were subjected to additional quality assessments, including evaluation of total base count, read number, GC content, and other fundamental statistics. Genome assembly was performed using SPAdes v3.15.1, employing a multi-*k*-mer approach, while Jellyfish v2.2.10 was used for *k*-mer frequency analysis to estimate genome size, coverage, and heterozygosity. The final assembly, generated using a De Bruijn graph-based algorithm, yielded a total of 18,087,184 high-quality reads, with a GC content of 44.51% and a Q30 score of 92.79%. Assembly validation was conducted through read mapping and BUSCO analysis, resulting in 24 contigs with a cumulative genome size of 2,322,326 bp and an N50 value of 205,025 bp.

### 2.2 Taxonomy and phylogenetic relationship

Following the assembly of the complete or draft genome, BLAST analysis was performed to determine the taxonomic identity of each scaffold by assessing sequence similarity. The best hit and the top five hits for each scaffold were identified using the NCBI NT database with ANI (Average Nucleotide Identity) values ≥ 95–96% confirming species identity (Richter and Rosselló-Móra, 2009). Supplementary Table S1 summarizes the top BLAST hits for the five longest contigs, ranked by sequence length. A genome map was generated using Proksee server (Grant et al., 2023). The genome sequence of Cys2-2 was submitted to the Type (Strain) Genome Server (TYGS) for taxonomic classification and phylogenetic analysis (Meier-Kolthoff and Göker, 2019). Closest type strains were identified using the MASH algorithm, followed by refined distance calculations using the Genome BLAST Distance Phylogeny (GBDP) method with the “coverage” algorithm (Meier-Kolthoff et al., 2013). Phylogroup assignment was performed using EzClermont (Waters et al., 2020).

### 2.3 Genome feature, gene prediction, functional annotation and safety

Structural genome annotation of UTNCys2-2 was performed using PROKKA v1.14.5 (Seemann, 2014). Coding sequences were predicted with Prodigal (Hyatt et al., 2010), rRNA genes with RNAmmer, and tRNA and tmRNA genes with Aragorn (Laslett and Canback, 2004). Signal peptides were identified using SignalP (Teufel et al., 2022), and noncoding RNAs were detected with Infernal (Nawrocki and Eddy, 2013). Functional annotation was carried out using InterProScan and EggNOG (Huerta-Cepas et al., 2019). Moreover, the identification of antibiotic resistance genes was performed using the Comprehensive Antibiotic Resistance Database (CARD) (Jia et al., 2017) in conjunction with the Resistance Gene Identifier (RGI) tool (Zankari et al., 2012). To further detect acquired antimicrobial resistance genes and chromosomal point mutations, ResFinder v4.1 (Bortolaia et al., 2020) was employed with a sequence identity threshold of 90% and a minimum alignment length of 60%. Prediction of putative virulence factors was carried out using the Virulence Factors Database (VFDB) (Liu et al., 2019), and bacterial pathogenicity potential was assessed through the PathogenFinder web server (Cosentino et al., 2013). The identification of prophage regions within the UTNCys2-2 genome was conducted using the PHAge Search Tool Enhanced Release (PHASTER) (Arndt et al., 2016). To detect CRISPR arrays, associated Cas genes, and potentially incomplete Cas elements, CRISPRCasFinder version 4.2.20 was employed (Couvin et al., 2018).

### 2.4 Carbohydrate-active enzyme (CAZyme) annotation

CAZyme annotation in UTNCys2-2 was performed using the dbCAN2 meta server (Zhang et al., 2018). This platform integrates multiple complementary algorithms to enhance the accuracy of carbohydrate-active enzyme prediction, including DIAMOND for fast sequence alignment, HMMER utilizing HMMdb v7 for hidden Markov model-based domain identification, and Hotpep for short, conserved motif detection.

### 2.5 KEGG and MetaCyc pathway analysis

To explore the potential involvement of predicted genes from the UTNCys2-2 strain in various biological pathways, the genes were mapped to canonical reference pathways using the KEGG (Kyoto Encyclopedia of Genes and Genomes) database through a custom analysis provided by Macrogen (Korea). The KEGG Orthology (KO) database for prokaryotes served as the reference framework for pathway mapping (Nakaya et al., 2013). Additionally, the MetaCyc analysis tool^1^ was employed to predict the participation of Cys2-2 genes in different metabolic pathways.

### 2.6 Prediction of secondary metabolites

The genomic contigs in FASTA format were analyzed using the Antibiotics and Secondary Metabolite Analysis Shell (antiSMASH) version 6.0.1 for the prediction of biosynthetic gene clusters (BGCs) associated with secondary metabolite production (Blin et al., 2021). As part of the “Subcluster Blast” module, the identified BGCs were queried against a specialized database comprising operons implicated in the biosynthesis of common secondary metabolite precursors, such as nonproteinogenic amino acids. Concurrently, the “KnownClusterBlast” module facilitated comparative analysis against the MIBiG repository, a curated database of experimentally characterized BGCs, to assess the similarity and potential novelty of the predicted clusters.

### 2.7 Pangenome analysis

Pangenome analysis was performed using Roary v1.007001 (Page et al., 2015) with MAFFT v7.427 (Katoh and Standley, 2013) for gene clustering based on complete protein-coding sequences, classifying genes into core (hardcore and softcore) and accessory (shell and cloud) genomes. Genomic data from nine *Weissella confusa* strains and one phylogenetically related *Leuconostoc* strain, included as an outgroup for comparative context, were retrieved from the NCBI database (Supplementary Table S2). The presence or absence of each gene cluster across all analyzed genomes is then determined, allowing for the identification of both shared and unique genetic elements. Based on their distribution across genomes, gene clusters are categorized into four groups: core genome (present in >99% of genomes), soft core genome (95–99%), shell genome (15–95%), and cloud genome (<15%). This classification provides insight into genetic conservation, evolutionary dynamics, and potential mechanisms of environmental adaptation within the species. During pan-genome analysis, genes with high sequence similarity are grouped into gene clusters.

### 2.8 Metabolite extraction

For metabolite recovery, bacterial cultures were grown in MRS medium at 37 °C for 24 h and subsequently harvested by centrifugation at 13,000 × g for 30 min at 4 °C. The resulting CFS was passed through 0.22 μm membrane filters (#STF020025H, Chemlab Group, United States) and stored at 4 °C until analysis. The extract was lyophilized and stored until further analysis.

### 2.9 LC–MS/MS analysis and metabolite identification

Untargeted metabolomic profiling was conducted as previously described (Molina et al., 2025). Briefly, lyophilized sample was reconstituted at a concentration of 1 mg/mL and centrifuged at 17,000 × g for 15 min at 4 °C prior to analysis. Clarified supernatant was analyzed using an AB SCIEX TripleTOF 5,600 + mass spectrometer (Sciex, Canada) coupled to a nanoACQUITY UPLC system (Waters, USA). Chromatographic separation was performed on an Eksigent 5C18-CL-120 column (300 μm × 150 mm) using a linear gradient of 5–80% acetonitrile containing 0.1% formic acid over 90 min, at a flow rate of 5 μL/min and a column temperature of 55 °C. Data acquisition was carried out in positive electrospray ionization (ESI+) mode under optimized source conditions. Untargeted acquisition employed SWATH-MS using 60 variable isolation windows, with MS1 scans acquired over an m/z range of 100–1,250 and MS2 scans over 100–2000. Accumulation times were set to 150 ms for MS1 and 30 ms for MS2. Ion source parameters were configured as follows: GS1, 15; GS2, 0; CUR, 25; TEM, 0; and ISVF, 5500 V. This low-temperature, low-nebulizer setup was optimized for micro-flow LC-ESI conditions, allowing efficient desolvation without auxiliary heating or GS2 desolvation gas, thereby minimizing in-source fragmentation and preserving thermolabile metabolites. Raw data were processed using MS-DIAL version 5.3.240719, and metabolite annotation was achieved by matching MS/MS spectra against MSP spectral libraries (https://systemsomicslab.github.io/compms/msdial/main.html#MSP; accessed 15 April 2025). MS-DIAL parameters included a retention time window of 1–90 min; MS1 and MS2 mass ranges of 100–1,250 Da and 100–2,000 Da, respectively; a minimum peak width of 5 scans; a minimum peak height of 1,000 (amplitude); a smoothing level of 3 scans; and an MS2 intensity cutoff of 10 (absolute amplitude). Mass slice width was set to 0.05 Da, with no exclusion mass list applied. Retention time tolerance was 0.1 min, while mass accuracy tolerances were 0.01 Da for MS1 and 0.05 Da for MS2. A minimum spectral similarity score of 70% was required for metabolite annotation, with additional mass tolerances of 0.01 Da (MS1) and 0.025 Da (MS2).

## 3 Results and discussion

### 3.1 Genomic characterization supports taxonomic assignment, genome stability, and biosafety of UTNCys2-2

**Figure 1:**
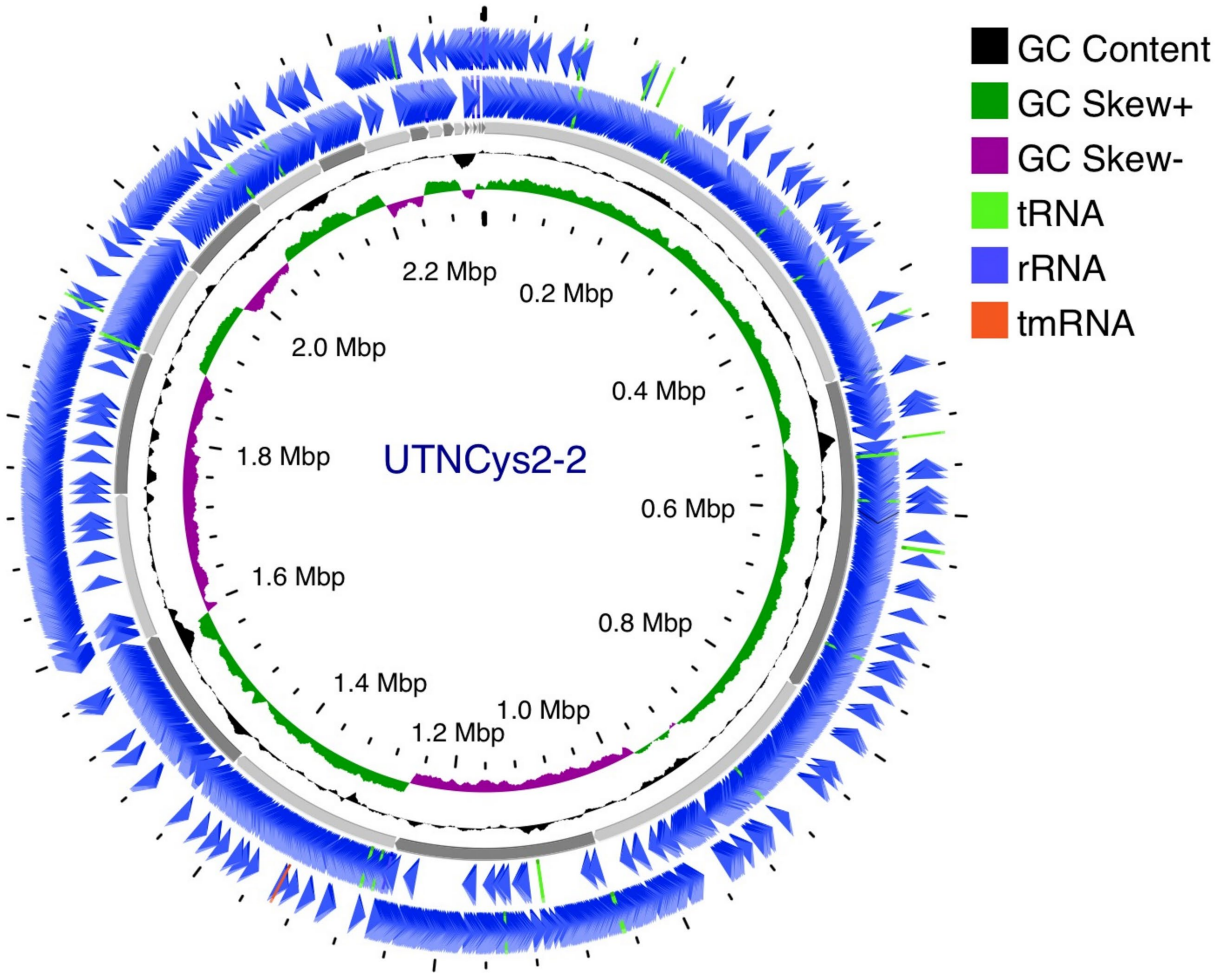
Genome view generated using Proksee server (https://proksee.ca). The contents are arranged in feature rings (starting with outermost ring): ring 1: Prokka annotation (+); ring 2: Prokka annotation (−); ring 3: backbone contigs; ORF: open reading frame; CDS (coding sequences) with Prokka annotation tRNA, rRNA, tmRNA are shown; ring 4: G + C content; ring 5: G/C skew (G + C/G − C).

**Figure 2:**
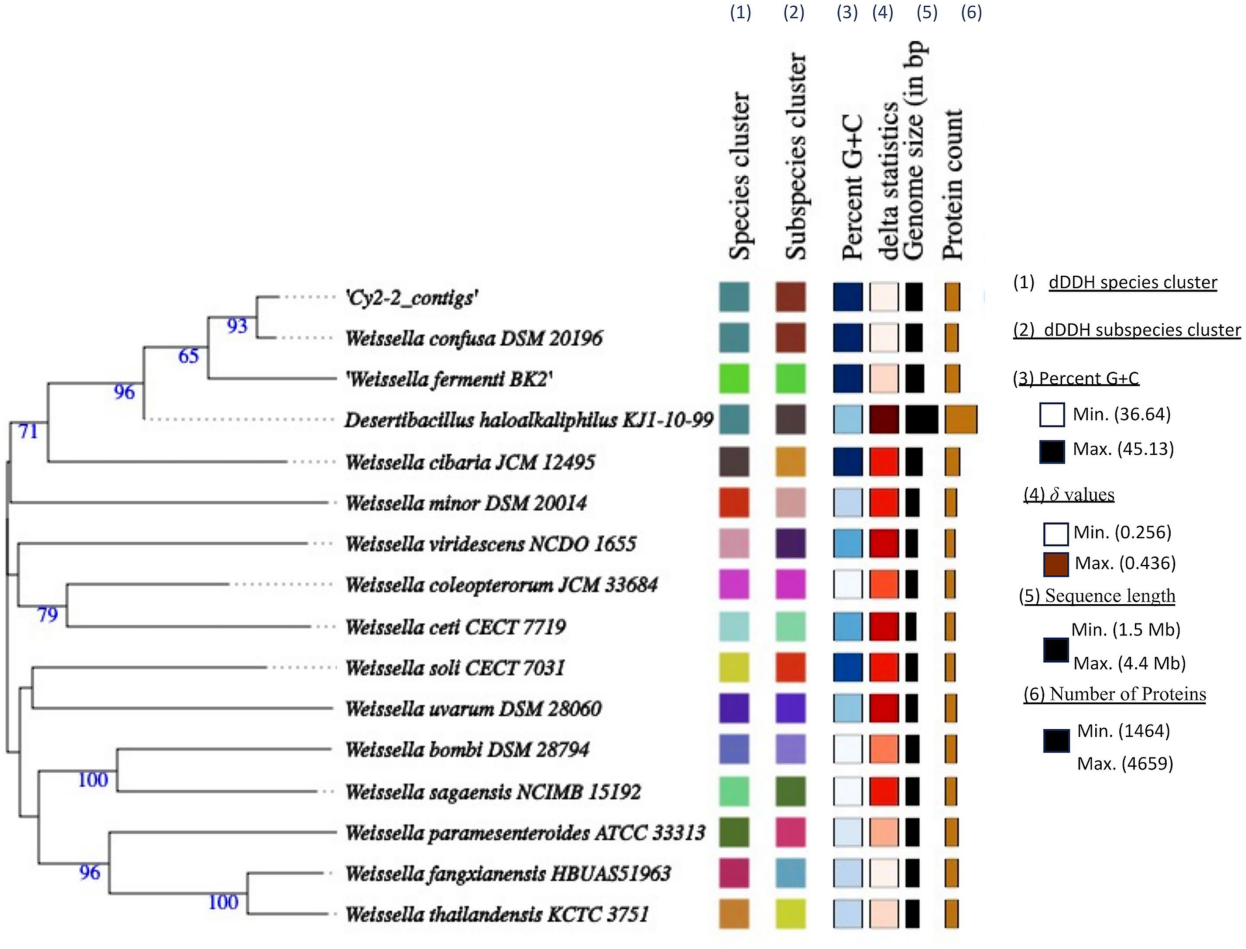
Phylogenetic tree based on TYGS results for the L1PEag1 whole-genome data set. Branch lengths are scaled in terms of GBDP (genome BLAST distance phylogeny method) distance; numbers below branches are GBDP pseudo-bootstrap support values from 100 replications. Leaf labels are annotated by affiliation to (1) species; (2) subspecies clusters; (3) genomic G + C content; (4) *δ* values; (5) overall genome sequence length; (6) number of proteins.

**Figure 3:**
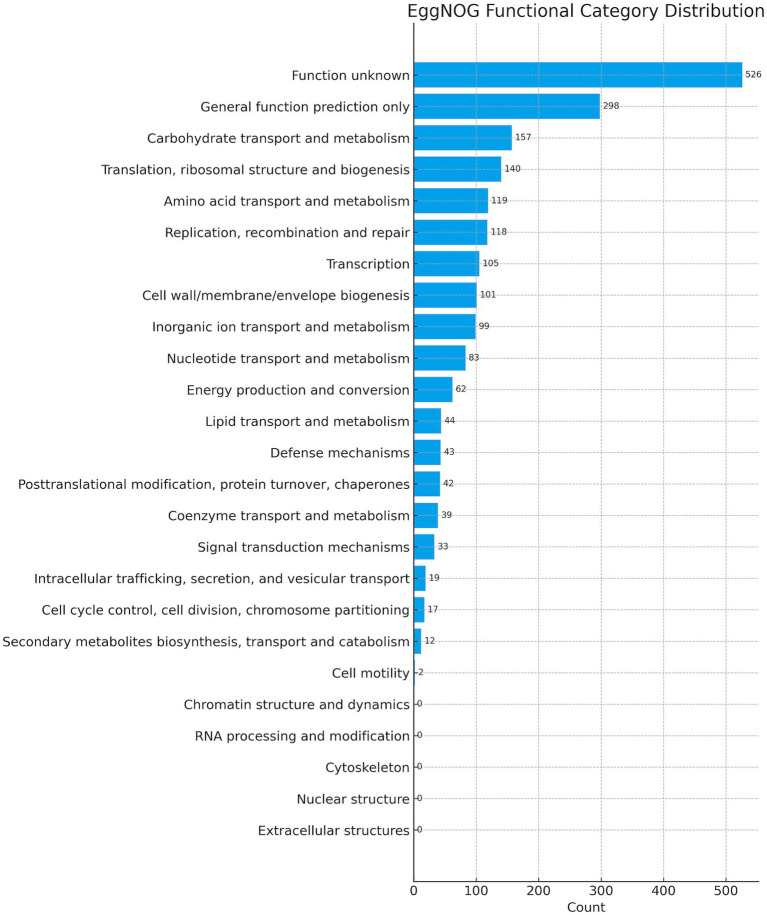
Distribution of predicted proteins across COG functional categories based on EggNOG annotation.

The complete genome of UTNCys2-2 consists of a single circular chromosome of 2,322,326 bp with a GC content of 44.59% and no detectable plasmids (Figure 1). These features are consistent with reported genome sizes (2.1–2.4 Mb) and GC contents (44–45%) for the *W. confusa* species (Thant et al., 2024). Although plasmids are present in some *Weissella* strains and have been associated with bacteriocin production or antimicrobial resistance, their absence in UTNCys2-2 may indicate reduced dependence on extrachromosomal elements for niche adaptation or stress tolerance (Fusco et al., 2015; Fusco et al., 2023; Singh et al., 2024). The genome encodes 2,194 protein-coding sequences, 76 tRNAs, and 10 rRNA genes, indicating a well-developed translational capacity (Table 1). While rRNA and tRNA gene copy numbers contribute to ribosome availability and codon decoding efficiency, overall translation efficiency also depends on codon usage bias, regulatory features of mRNA, and cellular physiological conditions (Klappenbach et al., 2000; Quax et al., 2015). Taxonomic identity was confirmed through multiple complementary approaches. BLAST alignment against the NCBI NT database and average nucleotide identity (ANI) analyses supported assignment within the *W. confusa* species (Supplementary Table S3). Whole-genome phylogenetic reconstruction placed UTNCys2-2 (Cys2-2_contigs) in a well-supported clade with *W. confusa* DSM 20196 (bootstrap value: 93%), clearly separated from other *Weissella* species such as *W. cibaria*, *W. minor*, and *W. viridescens* (Figure 2). Comparative genomic metrics, including GC content, genome size, coding potential, and delta statistics, further indicate a conserved genome architecture within the *W. confusa* cluster. Among the 2,194 predicted proteins, 2,040 were assigned to EggNOG functional categories (2,021 single EggNOG and 19 multi-EggNOG assignments), while 154 proteins lacked database matches, potentially representing strain-specific or novel functions (Figure 3).

**Table 1:** Genome features of UTNCys2-2.

Genome parameters	Values
Genome length (bp)	2,322,326
Plasmids	0
GC content (%)	44.59
Total number of genes	2,281
Coding genes	2,194
tRNA number of assembled genome	76
rRNA number of assembled genome	10
tmRNA number of assembled genome	1
CRISPR-Cas array*	2 (Cas-Type IIA)
Prophage (intact region)**	3
Antibiotic acquired genes***	None
Pathogenicity****	Nonhuman pathogen

* CRISPRFinder (https://crisprcas.i2bc.paris-saclay.fr/CrisprCasFinder/Index). **PHAge Search Tool Enhanced Release (PHASTER) (http://phaster.ca). ***ResFinder 4.1 (https://genepi.food.dtu.dk/resfinder); ****PathogenFinder (https://genepi.food.dtu.dk/pathogenfinder).

Genome annotation further revealed features indicative of enhanced genomic stability and defense against foreign DNA. UTNCys2-2 harbors a complete Type II-A CRISPR-Cas system, comprising core genes *cas*9, *cas*1, *cas*2, and *csn*2, along with two CRISPR arrays and one tmRNA. This system is known to provide adaptive immunity against bacteriophages and plasmids, thereby limiting HGT and contributing to long-term genome integrity (Ku et al., 2017). While CRISPR-Cas systems are widely distributed among lactic acid bacteria, the Type II-A subtype is relatively uncommon in *W. confusa*, suggesting a potentially unique evolutionary adaptation that may enhance ecological fitness and biosafety in this strain (Liu et al., 2025). In addition to CRISPR-mediated defense, three intact prophage regions were identified, along with several prophage-associated genes, including holins (Supplementary Figure S1). Prophage insertions have been reported to contribute to genome diversification, ecological competitiveness, and functional traits such as exopolysaccharide biosynthesis and antimicrobial activity in *W. confusa* and related LAB (Elshaghabee et al., 2017). While prophages and mobile genetic elements can promote genome plasticity, their presence in UTNCys2-2 appears balanced by robust genome surveillance mechanisms. Mobile elements detected include ISLsa1 (IS30 family) and ISSsu7 (IS110 family), which are associated with genome rearrangements and regulatory modulation (Partridge et al., 2018; Hiraizumi et al., 2024; Siddiquee et al., 2024). Importantly, no virulence genes or pathogenicity islands were detected, supporting the strain’s safety profile.

Antimicrobial resistance (AMR) screening revealed the presence of *vanY* and *vanT* genes within vanB/vanG-related clusters (Supplementary Figure S1) when analyzed using the CARD database, whereas these genes were not identified by Prokka annotation. This discrepancy highlights the importance of curated, domain-specific databases for accurate AMR detection, particularly when assessing strains for food or probiotic use (Seemann, 2014; Jia et al., 2017). Nonetheless, these resistance-associated genes were not linked to mobile genetic elements, substantially reducing the risk of horizontal dissemination. In compliance with European Food Safety Authority (EFSA) guidelines for food-grade microorganisms, no virulence determinants or transferable antimicrobial resistance (AMR) genes were detected in the UTNCys2-2 genome (EFSA Panel on Biological Hazards (BIOHAZ), 2021). Consistent with these findings, PathogenFinder classified UTNCys2-2 as a non-human pathogen with a confidence score of 99.93%, further supporting its biosafety for food and probiotic use. These genomic attributes define UTNCys2-2 as a taxonomically well-defined and genomically stable strain, characterized by the presence of adaptive immune defenses, moderate genome plasticity, and the absence of clinically relevant virulence factors or mobile resistance determinants. This genomic architecture supports its suitability for applications in food fermentation, probiotic development, and microbial biotechnology.

### 3.2 Comprehensive CAZyme repertoire highlights carbohydrate adaptability and probiotic potential of UTNCys2-2

**Figure 4:**
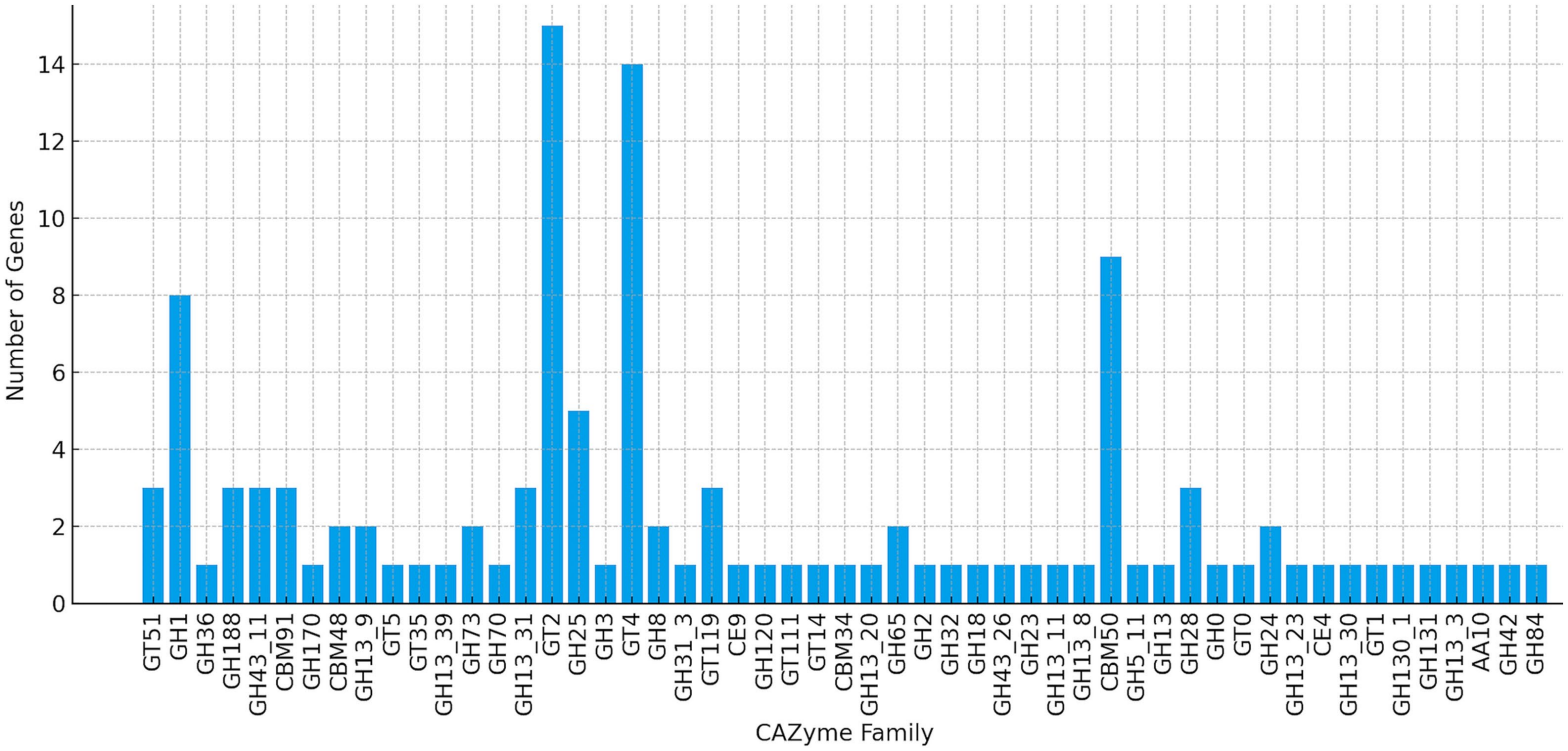
CAZyme distribution in UTNCys2-2 genome.

The CAZyme profile indicates a metabolically versatile organism with a functionally diverse set of enzymes, supporting adaptation to varied ecological niches. Genomic analysis identified 118 CAZyme-encoding genes, including 52 glycoside hydrolases (GHs), 41 glycosyltransferases (GTs), and 13 carbohydrate-binding modules (CBMs), reflecting the capacity to both degrade and synthesize a wide range of polysaccharides (Figure 4). GH families such as GH1, GH2, GH18, GH30, GH13_20, GH13_39, GH42, GH43_11, and GH65 are associated with oligosaccharide degradation, indicating the ability to utilize complex carbohydrates, including plant-derived substrates. The presence of GH70 enzymes, which catalyze the biosynthesis of *α*-glucans like dextran, aligns with previous observations in strains such as *W. confusa* WcL17 and WCP-3a (Hernández-Oaxaca et al., 2021). These enzymes, together with hemicellulose- and starch-degrading activities, indicate a finely tuned enzymatic toolkit tailored to carbohydrate-rich environments (Hernández-Oaxaca et al., 2021). Comparative genomics further positions *W. confusa* as a species with an enriched CAZyme arsenal relative to other members of the genus. While *W. confusa* and *W. cibaria* harbor on average 88 and 101 CAZyme genes, respectively, species like *W. viridescens* contain significantly fewer (approximately 21 genes), highlighting *W. confusa* evolutionary adaptation toward enhanced carbohydrate utilization (Wang M. et al., 2022; Wang Y. et al., 2022). Functionally, this extensive CAZyme repertoire implies significant biotechnological promise. GTs and GHs support efficient utilization of complex plant-derived carbohydrates and is consistent with previous reports of floral and plant-fermenting *Weissella* strains that play key roles in oligosaccharide degradation and EPS production. The identification of a GH70 family gene, typically associated with EPS synthesis, and a GT2-type glycosyltransferase, further supports the strain ability to interact with host surfaces, form biofilms, or modulate microbial communities in floral ecosystems. The ability of UTNCys2-2 to synthesize EPS via GH70 and GT2 family enzymes may contribute not only to textural and rheological properties in fermented foods but also to potential health benefits. EPS have been associated with probiotic traits such as reinforcement of gut epithelial barrier integrity and inhibition of pathogenic colonization (Hernández-Oaxaca et al., 2021; Wang M. et al., 2022; Wang Y. et al., 2022). However, the CAZyme landscape of *W. confusa* UTNCys2-2 supports its classification as a functionally robust and biotechnologically attractive strain, with potential applications in food fermentation and development of functional probiotic formulations.

### 3.3 Enriched pathways highlight robust EPS biosynthesis and stress tolerance associated with probiotic and fermentative performance

**Figure 5:**
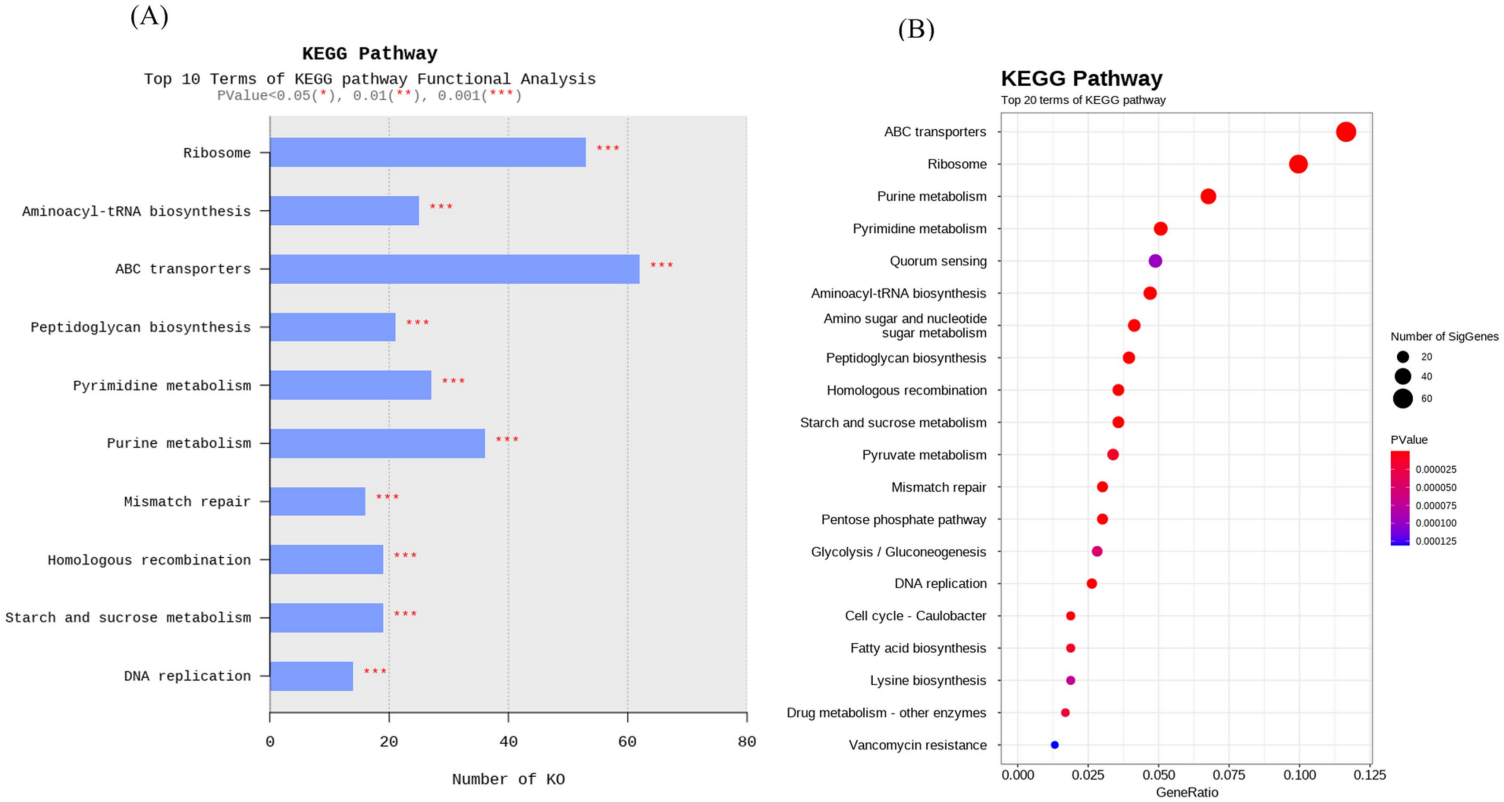
KEGG pathway enrichment analysis of significant annotated genes. **(A)** Dot plot showing the top 20 enriched KEGG pathways based on GeneRatio (proportion of genes mapped to each pathway), *p*-value (color gradient), and number of significant genes (dot size). **(B)** Bar plot displaying the top 10 pathways based on the absolute number of KEGG Orthologs (KOs) annotated. Statistical significance is denoted by asterisks, where (*p* < 0.05), (*p* < 0.01), and (*p* < 0.001).

The KEGG pathway enrichment analysis reveals complementary insights into the functional potential of the UTNCys2-2 strain. The dot plot highlights the top 20 pathways ranked by gene ratio, *p*-value, and the number of significant genes, while the bar plot emphasizes the top 10 pathways based on the total number of annotated KEGG Orthologs (KOs) (Figures 5A,B). Despite their different ranking metrics, both plots consistently identify key metabolic and cellular processes. Enriched pathways included ABC transporters, ribosome biogenesis, and aminoacyl-tRNA biosynthesis, indicating efficient nutrient uptake, protein synthesis, and cellular homeostasis. The enrichment of purine and pyrimidine metabolism, peptidoglycan biosynthesis, and DNA replication and repair pathways supports active nucleotide turnover, robust cell wall formation, and genomic stability associated with probiotic and fermentative bacteria (Wang M. et al., 2022; Wang Y. et al., 2022; Tan et al., 2022). Additional enrichment of carbohydrate and lipid metabolism, quorum sensing, cell cycle regulation, and vancomycin resistance pathways reflects metabolic versatility, coordinated cellular regulation, and potential antimicrobial resilience in gastrointestinal and fermented food environments (Hernández-Oaxaca et al., 2021; Fanelli et al., 2022; Fhoula et al., 2022). The capacity of UTNCys2-2 for EPS production is supported by the enrichment of KEGG pathways involved in carbohydrate metabolism and sugar nucleotide biosynthesis, including starch and sucrose metabolism (KO00500) (Supplementary Figure S2) and amino sugar and nucleotide sugar metabolism (KO00520) (Supplementary Figure S3), which encode enzymes for the generation of activated sugar donors such as UDP-glucose and UDP-galactose required for EPS synthesis (Falasconi et al., 2020). The presence of the pentose phosphate pathway (KO00030) and glycolysis/gluconeogenesis (KO00010) further supports EPS biosynthesis by providing NADPH, ribose-5-phosphate, metabolic intermediates, and energy (Freitas et al., 2011; Zeidan et al., 2017). Additionally, enrichment of ABC transporters (KO02010) and protein export pathways (KO03060) suggests mechanisms for EPS translocation and extracellular assembly (Netrusov et al., 2023). Together, these pathways demonstrate a genomic framework supporting efficient EPS biosynthesis and secretion, consistent with the strains functional role in fermented food systems (Tian et al., 2025).

Moreover, genome annotation using Prokka revealed the presence of several genes involved in the glycogen biosynthesis pathway. Glycogen, a major water-soluble polysaccharide, is widely distributed among bacteria, archaea, and eukaryotes, where it plays a crucial role in cellular physiology (Sharma et al., 2023). Under unfavorable conditions, glycogen is degraded to support prolonged bacterial survival. In this study, a conserved and functional glycogen biosynthesis operon (*glg* operon) was identified in UTNCys2-2. This pathway encompasses key enzymes, including phosphoglucomutase (*pgm*), glucose-1-phosphate adenylyltransferase (*glg*C and *glg*D), ADP-glucose-specific glycogen synthase (*glg*A), and a branching enzyme (*glg*B). In addition, amylopullulanase (EC 3.2.1.41), an extracellular enzyme identified in Weissella species was annotated. These enzyme plays a pivotal role in carbohydrate metabolism by enabling the utilization of diverse polysaccharides present in plant-based substrates (Fusco et al., 2023). This enzymatic activity not only supports bacterial growth in carbohydrate-rich environments but also contributes to the production of EPS, which enhance the texture and stability of fermented foods (Sharma et al., 2023). The presence of amylopullulanase in Weissella strains underscores their potential in food biotechnology, especially in the development of functional fermented products with improved rheological properties. For instance, the dextran-producing capabilities of have been associated with its amylopullulanase activity, leading to the synthesis of oligosaccharides like panose, which possess prebiotic attributes and contribute to the health benefits of fermented beverages (Wang M. et al., 2022; Wang Y. et al., 2022).

**Figure 6:**
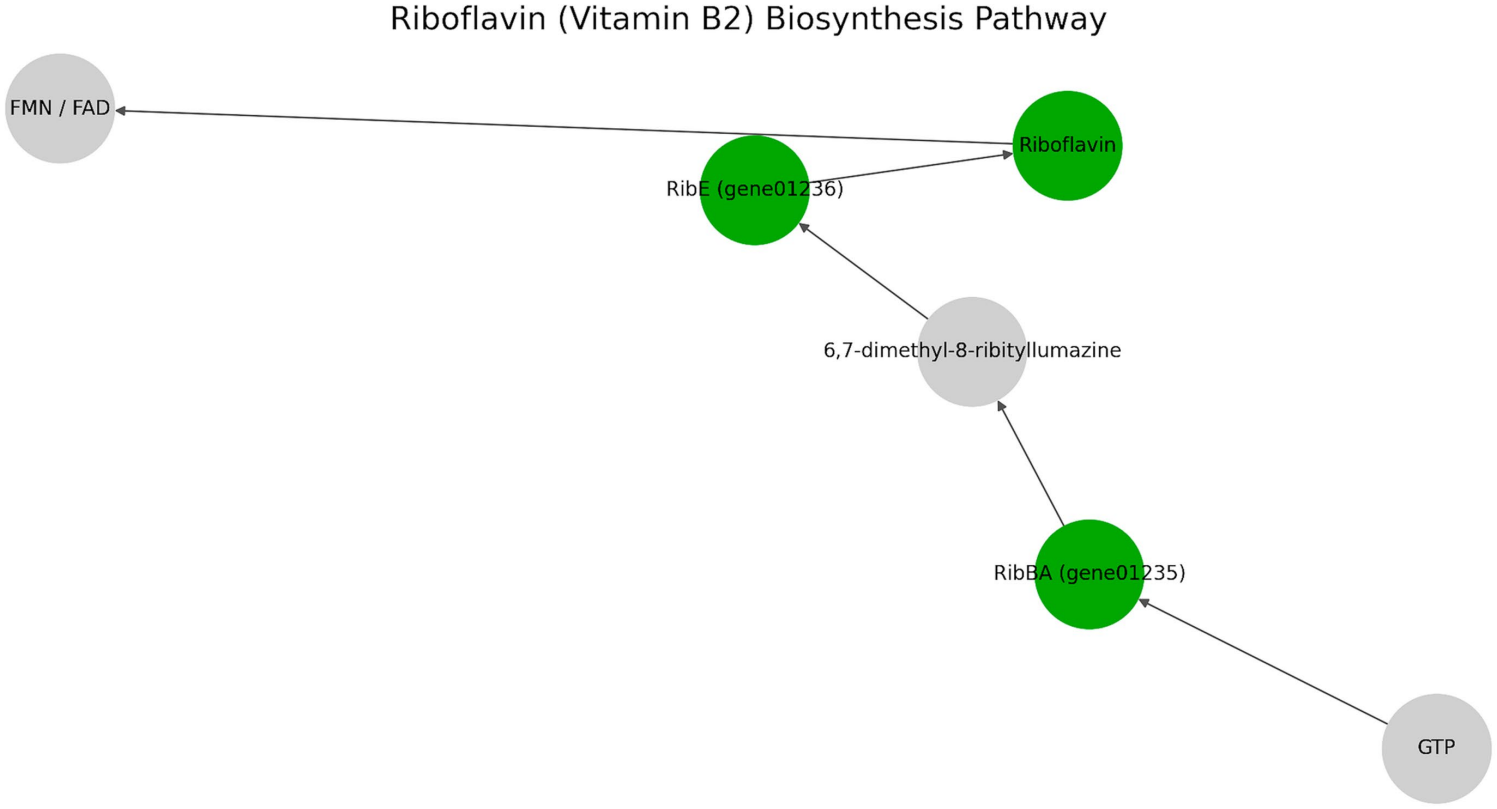
Riboflavin (vitamin B2) biosynthesis pathway. The diagram illustrates the key steps in the riboflavin biosynthetic pathway, beginning with GTP and ending in riboflavin and its coenzyme derivatives (FMN/FAD). Green nodes represent genes detected in the UTNCys2-2 genome (e.g., *ribBA*, *ribE*), highlighting the strain’s capacity to synthesize riboflavin *de novo*. Gray nodes indicate intermediate metabolites or steps.

Furthermore, MetaCyc annotation of the UTNCys2-2 genome reveals key traits supporting its probiotic potential and suitability for food fermentation (Supplementary Table S4). Key enzymes like UDP-glucose 4-epimerase and UTP-glucose-1-phosphate uridylyltransferase support UDP-sugar biosynthesis, essential for EPS assembly. The strain exhibits robust carbohydrate metabolism, including the heterofermentative phosphoketolase pathway and diverse sugar-modifying enzymes (e.g., glycosyltransferases, maltose phosphorylase), underpinning its capacity for EPS biosynthesis (Fusco et al., 2015). EPS-related genes, along with teichoic acid biosynthesis pathways, suggest strong potential for biofilm formation, host adhesion, and immune modulation, crucial for gut colonization and functional food applications (Weidenmaier and Peschel, 2008). Genes involved in amino acid and cofactor biosynthesis further support its adaptability in nutrient-limited or dynamic environments. Even more, the presence of key genes involved in the *de novo* biosynthesis of riboflavin (vitamin B2), highlighting its potential as a natural vitamin-producing strain. Specifically, genes such as *rib*BA, *rib*E, and *rib*C were identified, encoding enzymes critical for the conversion of GTP and ribulose-5-phosphate into riboflavin (Figure 6). This pathway includes the formation of intermediates like 6,7-dimethyl-8-ribityllumazine, catalyzed by *rib*E, and the final synthesis of riboflavin via riboflavin synthase (*rib*C). The presence of this complete pathway suggests that UTNCys2-2 can synthesize riboflavin autonomously, enhancing the nutritional profile of fermented products without external vitamin fortification. Moreover, riboflavin contributes to probiotic fitness by supporting oxidative stress tolerance through its coenzyme derivatives FMN and FAD (Ashoori and Saedisomeolia, 2014). It also plays a role in host immune modulation, particularly through the activation of mucosal-associated invariant T (MAIT) cells, which are influenced by riboflavin derivatives produced by gut microbiota (Fang et al., 2025). These features make *W. confusa* UTNCys2-2 a promising candidate for next-generation probiotic applications.

### 3.4 Genome mining reveals T3PKS cluster with potential for novel secondary metabolites

**Figure 7:**
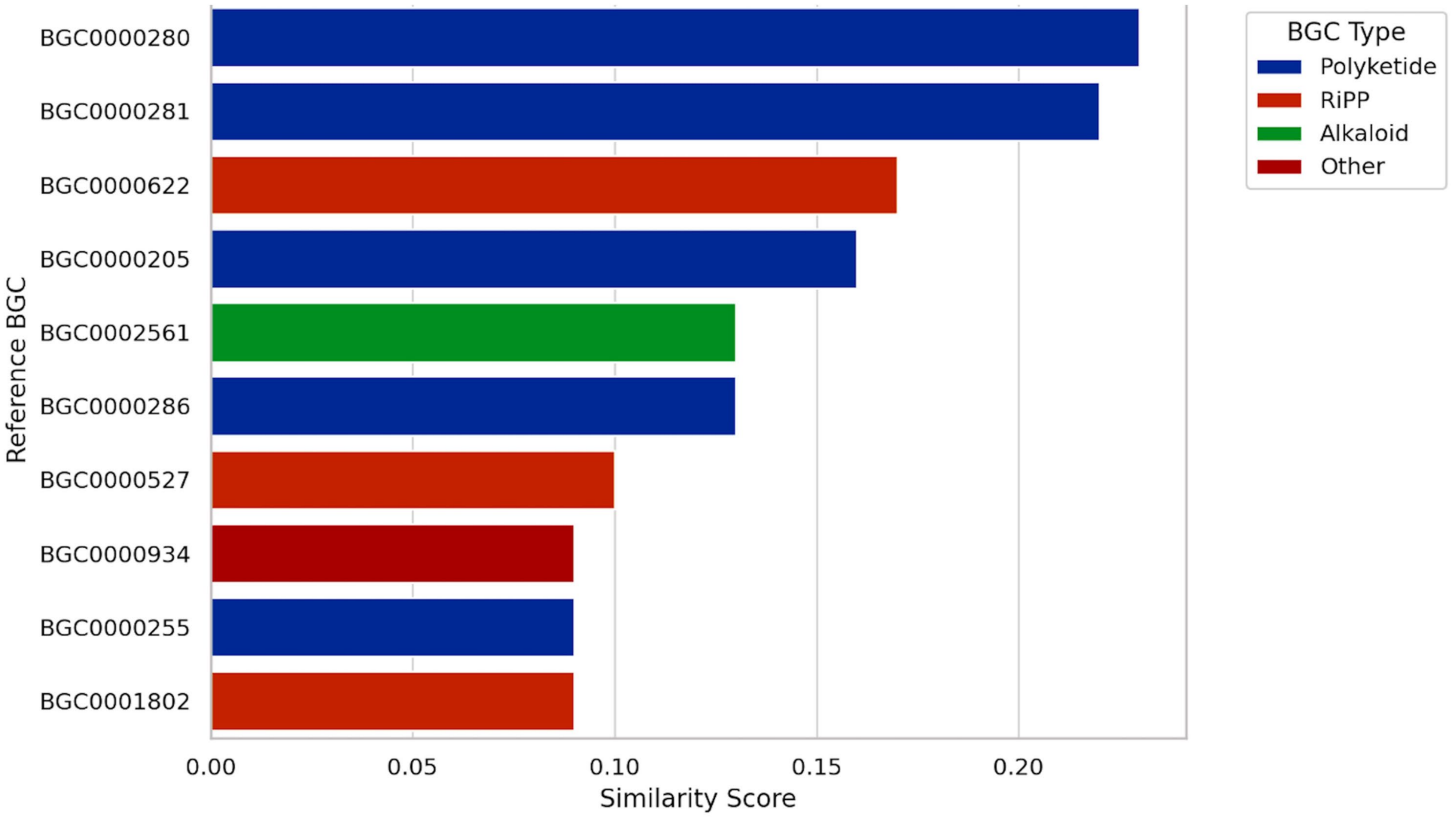
Bar graph visualizing the similarity scores of the T3PKS BGC of UTNCys2-2 against known reference clusters. Each bar represents a reference BGC, colored by its biosynthetic type (polyketide, RiPP, alkaloid, etc.). The graph highlights that the highest similarity is observed with two polyketide-producing BGCs from *Pseudomonas fluorescens* (BGC0000280 and BGC0000281), with similarity scores of 0.23 and 0.22, respectively. BGC0000622: *Bacillus megaterium*; BGC0000205: *Candidatus Endobugula sertula*; BGC0002561: *Streptomyces* sp.; BGC0000286: *Streptomyces* sp. KO-3988; BGC0000527: *Bacillus* sp. HIL-Y85/54728; BGC0000934: *Kitasatospora* sp. HKI 714; BGC0000255: Uncultured bacterium; BGC0001802: *Streptomyces malaysiense*.

Genome mining identified a T3PKS biosynthetic gene cluster showing low to moderate similarity (0.09–0.22) to characterized clusters in the MIBiG database (Figure 7). The closest matches corresponded to polyketide clusters BGC0000280 and BGC0000281, associated with 2,4-diacetylphloroglucinol biosynthesis in *Pseudomonas fluorescens*, along with additional similarities to RiPP (ribosomally synthesized and post-translationally modified peptides), and alkaloid BGCs, including BGC0000622 (RiPP) and BGC0002561 (alkaloid). Comparative analyses indicate that *W. confusa* genomes typically harbor a limited, conserved BGC repertoire dominated by T3PKS clusters (Yuan et al., 2021), consistent with the profile observed in UTNCys2-2. Previous studies have reported bioactive compound production and related BGCs in *W. confusa* strains, including antimicrobial metabolites in WM36 (Pelyuntha et al., 2020) and T3PKS-, RiPP-, and aryl polyene-associated clusters in strains W1 and W2 (Thant et al., 2024). These findings may explain the antimicrobial activity against *E. coli* and *Salmonella* (Tenea and Lara, 2019). Altogether, the detection of a T3PKS cluster in UTNCys2-2 with multiple similarity links to known metabolite classes suggests a potentially novel biosynthetic capability. Such secondary metabolites are frequently associated with ecological fitness traits, including antimicrobial activity, modulation of quorum sensing, and niche-specific chemical communication (Hertweck, 2009). The low sequence similarity of this T3PKS cluster to characterized biosynthetic pathways suggests a potentially novel metabolic route, plausibly shaped by adaptive responses to the complex and chemically dynamic floral environment from which the strain was isolated (Cimermancic et al., 2014; Donia and Fischbach, 2015). Further studies integrating transcriptomic or proteomic profiling, targeted metabolite isolation, and functional genetic validation will be essential to link this T3PKS cluster to specific polyketide products and to clarify its contribution to antimicrobial activity and biotechnological potential. Combined with its genomic plasticity and metabolic versatility, this feature highlights the strain’s promise as a source of novel bioactive compounds for diverse biotechnology applications.

### 3.5 Pangenome analysis reveals strain-specific genetic diversity and specialized carbohydrate metabolism in UTNCys2-2

**Figure 8:**
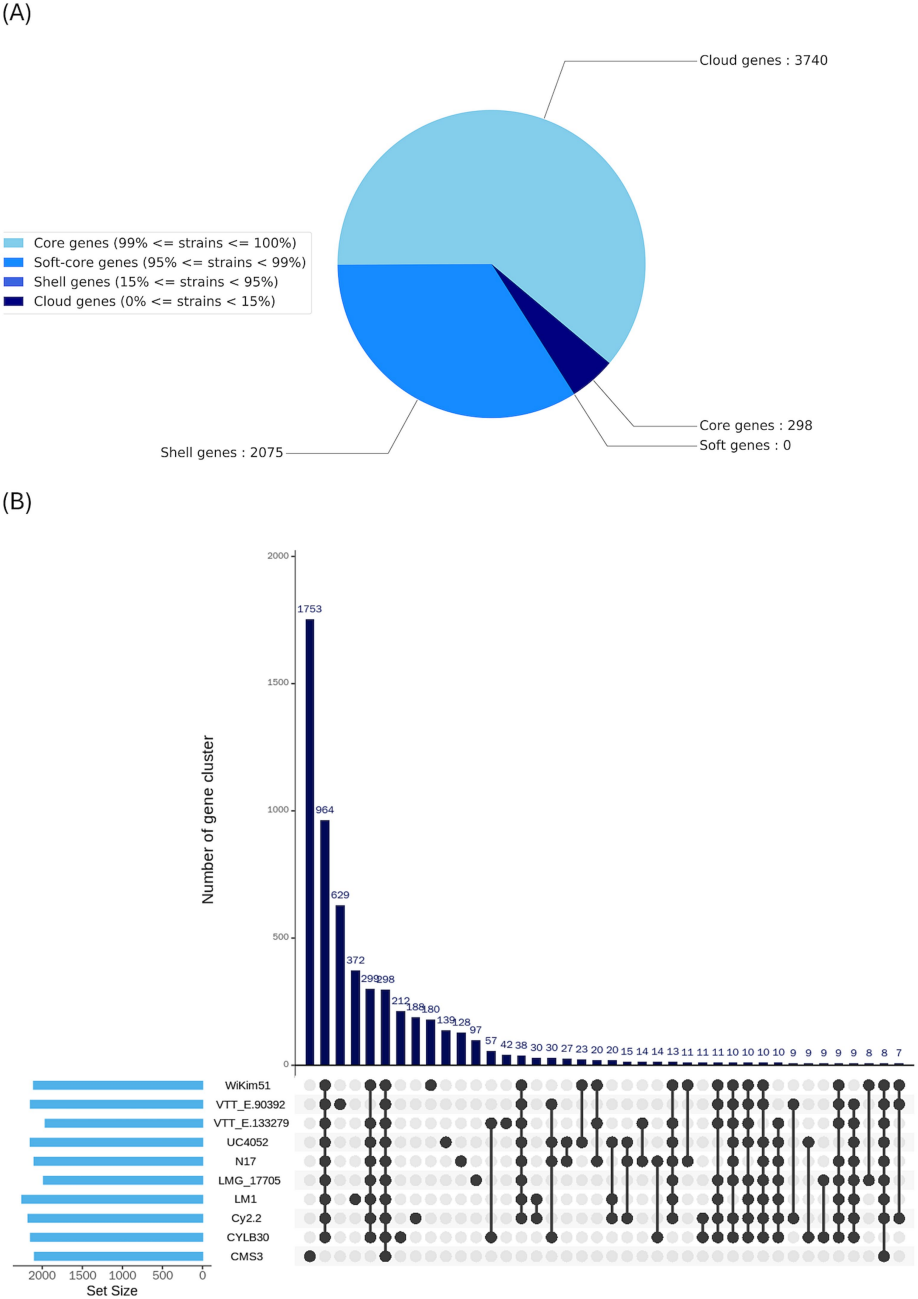
Pangenome analysis. **(A)** Pie chart illustrates the distribution of genes within the UTNCys2-2 genome across the pangenome categories: core, soft core, shell, and cloud; **(B)** UpSet plot comparing the distribution of gene clusters across multiple *W. confusa* strains and *Leuconostoc*. The top bar chart shows the number of unique or shared gene clusters across specific strain combinations, while the left-side bar chart represents the total gene cluster count (set size) per strain.

Pangenome analysis revealed that UTNCys2-2 forms a genetically distinct lineage relative to the other nine analyzed strains. The pie chart summarizes the distribution of gene clusters across the pangenome, classified into core, shell, and cloud categories (Figure 8A). Of the total gene clusters, 298 were identified as core genes shared by all strains, reflecting a compact conserved genome associated with essential cellular functions. No soft-core genes (present in 95–99% of strains) were detected, indicating a sharp separation between universally conserved and variably distributed genes. In contrast, a large proportion of genes belonged to the shell (2,075) and cloud (3,740) fractions, with cloud genes predominating (Supplementary Figure S3), indicative of an open pangenome and extensive strain-level genetic diversity. This pattern is further supported by the UpSet plot analysis (Figure 8B), which shows that UTNCys2-2 contributes a substantial number of unique gene clusters not shared with other strains, highlighting pronounced genomic plasticity and niche-specific adaptation that may underpin its probiotic and biotechnological potential.

Gene presence-absence analysis identified several UTNCys2-2-specific genes involved in carbohydrate and glycan metabolism, indicating specialized polysaccharide biosynthetic capabilities. The genome encodes UDP-glucose 4-epimerase, which enhances intracellular galactose availability and supports EPS biosynthesis. In addition, genes encoding UDP-linked glycosyltransferases, including UDP-N-acetylgalactosamine–undecaprenyl-phosphate N-acetylgalactosamine phosphotransferase (*mur*G) and UDP-Gal:*α*-D-GlcNAc-diphosphoundecaprenol *β*-1,3-galactosyltransferase (*wbb*D), were annotated with EggNOG. These enzymes are characteristic of Wzx/Wzy-dependent heteropolysaccharide biosynthetic pathways and are essential for glycan initiation, repeat-unit elongation, and cell wall polysaccharide diversification (Islam and Lam, 2014). Such polysaccharide structures are associated with probiotic-related traits, including biofilm formation, epithelial adhesion, and immunomodulatory interactions (Jurášková et al., 2022). In parallel, the presence of GH70 family enzymes indicates genetic potential for dextransucrase-mediated dextran synthesis via a sucrose-dependent extracellular pathway, as previously reported in *W. confusa* (Hernández-Oaxaca et al., 2021; Wang M. et al., 2022; Wang Y. et al., 2022). Together, these findings suggest that UTNCys2-2 harbors distinct and mechanistically independent EPS biosynthetic systems, enabling the production of both dextran-type homopolysaccharides and structurally diverse heteropolysaccharides, with EPS composition likely influenced by carbon source availability. This genomic prediction is supported by experimental evidence demonstrating robust EPS production by UTNCys2-2 under different carbohydrate conditions (Ascanta et al., 2025). Additional genes contributing to cell surface architecture and metabolic adaptability were also identified. The detection of glycerol-3-phosphate cytidylyltransferase, a key enzyme in wall teichoic acid biosynthesis, suggests roles in maintaining peptidoglycan integrity, modulating autolytic activity, and facilitating host–microbe interactions. Furthermore, the presence of 2-dehydro-3-deoxy-D-gluconate 5-dehydrogenase, involved in myo-inositol metabolism and NADPH regeneration, links carbohydrate utilization to redox balance and stress response (Gupta et al., 2017; Lahmar et al., 2024). The presence of EPS, teichoic acid, and inositol metabolism genes reflects UTNCys2-2 multifunctionality and potential for probiotic and carbohydrate-based applications.

### 3.6 Metabolomic profile of UTNCys2-2 cell-free supernatant indicates metabolic diversity

Untargeted LC–MS/MS analysis was conducted to qualitatively profile metabolites produced under defined culture conditions rather than to perform differential abundance testing, and identified compounds were annotated based on accurate mass, fragmentation patterns, and database matching. Thus, metabolomic profiling of the CFS revealed a broad spectrum of bioactive metabolites encompassing alkaloids, peptides, functional carbohydrates, and phenolic compounds (Table 2), indicating that the functional potential of UTNCys2-2 is supported by a chemically diverse metabolite repertoire. In line with genomic analysis, no specific bacteriocins were detected by untargeted LC–MS/MS, suggesting the capacity to produce novel or poorly characterized secondary metabolites whose expression may be conditional or below current detection limits. Among the detected compounds, indole-derived alkaloids such as 1,2,3,4-tetrahydro-6-methoxy-1-oxo-*β*-carboline were identified. Although antimicrobial activity of this specific metabolite was not experimentally assessed in this study, β-carbolines as a chemical class are well documented for antimicrobial effects (Yu et al., 2025). Similar alkaloid-associated metabolites have been reported in *Weissella* and other LAB isolated from coffee-associated environments, where they are proposed to contribute to ecological competitiveness in complex microbial communities (Cifuentes et al., 2025). The CFS also contained small bioactive peptides, including the proline-rich hexapeptide Val-Leu-Pro-Val-Pro-Gln and the hydrophobic dipeptide N-leucyl-leucine, reflecting the proteolytic activity of UTNCys2-2. Proline-rich and hydrophobic peptides have been increasingly associated with antimicrobial activity in probiotic LAB isolated from fermented coffee cherries, acting through interactions with bacterial membranes or ribosomal targets (Cifuentes et al., 2025). In addition to antimicrobial-related metabolites, several compounds with relevance to host interaction were detected. L-tryptophan, a precursor of indole signaling molecules, plays a key role in immune modulation and gut homeostasis via aryl hydrocarbon receptor–mediated pathways, a mechanism commonly linked to probiotic function (O’Mahony et al., 2015). Functional disaccharides such as melibiose and gentiobiose further support a potential prebiotic effect by selectively promoting beneficial gut microbiota and contributing to microbial community stability (Song, 2024). The presence of phenolic metabolites, including mulberrin and methoxylated flavones, suggests the ability of UTNCys2-2 to biotransform plant-derived substrates into antioxidant and anti-inflammatory compounds, a trait reported for plant-associated LAB (Fusco et al., 2015). Phenylethanolamine, a low-molecular-weight biogenic amine detected in the CFS, represents a common microbial metabolite that is generally well tolerated at low concentrations and efficiently metabolized by host amine oxidases (Saha Turna et al., 2024). Consistent with this multifactorial metabolite profile, UTNCys2-2 exhibits concentration-dependent cytotoxicity toward NCTC murine fibroblast cells, while maintaining good biocompatibility at lower concentrations (Ascanta et al., 2025). Taken together, these results indicate that the antimicrobial and probiotic properties of *W. confusa* UTNCys2-2 are not attributable to a single dominant compound or classical bacteriocin but rather emerge from a coordinated network of secondary metabolites with complementary antimicrobial, metabolic, and host-interactive functions.

**Table 2:** Chemical classes and reported biological activities of metabolites identified in UTNCys2-2.

Chemical class	Compound	Reported bioactivity*
Carbohydrates (Disaccharides)	Melibiose	Prebiotic substrate; supports probiotic growth (Rastall and Gibson, 2015)
	Gentiobiose	Prebiotic effect; fermentable by beneficial gut microbes (Hutkins et al., 2016)
Amino acids and Peptides	L-Tryptophan	Boosts gut immunity and microbiota through AhR and its indole derivatives, which have antimicrobial activity (Roager and Licht, 2018).
	N-Leucyl-leucine	Dipeptide with weak antimicrobial reports (Corrêa et al., 2023).
	Val-Leu-Pro-Val-Pro-Gln	Small bioactive peptide; possible antimicrobial effects (Molina et al., 2025; Cifuentes et al., 2025)
Biogenic amines and related nitrogenous metabolites	Phenylethanolamine	Microbial metabolic product; involved in microbial competition and host signaling (Linares et al., 2011)
Glycation/Maillard reaction products	N-deoxyfructosylleucylisoleucine	Metabolic by-product; limited antimicrobial activity (García et al., 2022)
Alkaloids	1,2,3,4-tetrahydro-6-methoxy-1-oxo-β-carboline	Antimicrobial activity reported for β-carbolines; DNA intercalation and enzyme inhibition (Yu et al., 2025)
	Reserpine	Antimicrobial and resistance-modulating activity (Lomovskaya et al., 1999)
Terpenoids and Terpenoid derivatives	Kobusone (sesquiterpenoid)	Antibacterial and antifungal activity (Singh and Sharma, 2015)
	Ganolactone B	Antimicrobial and anti-inflammatory activity (Galappaththi et al., 2022)
	Pseudo-anisatin	Similarity to various cyanobactins; promising pharmaceutical compound (Sivonen et al., 2010)
Triterpenoid/Steroidal glycosides	(2S,3R,4S,5R)-2-[[(1R,3S,4S,5S,7R,9S,12R,14S,17R,18R,19R,21R,22S)-5-hydroxy-22-(2-hydroxypropan-2-yl)-3,8,8,17,19-pentamethyl-23,24-dioxaheptacyclo[19.2.1.01,18.03,17.04,14.07,12.012,14]tetracosan-9-yl]oxy]oxane-3,4,5-triol	Not investigated
	Ergosta-5,22,25-trien-3-ol (3S,8S,9S,10R,13R,14S,17R)-17-[(2R,3E,5S)-5,6-dimethylhepta-3,6-dien-2-yl]-10,13-dimethyl-2,3,4,7,8,9,11,12,14,15,16,17-dodecahydro-1H-cyclopenta[a]phenanthren-3-ol	Antifungal and antibacterial sterol activity (Sang et al., 2021)
Polycyclic terpenoids/Meroterpenoids	(1R,2R,5R,7R,10S,11S,14S,16S,19R,20R,23R,25R,28S,29S,32S,34S)-5-Ethyl-2,11,14,20,23,29,32-heptamethyl-4,13,22,31,37,38,39,40-octaoxapentacyclo[32.2.1.17,10.116,19.125,28]tetracontane-3,12,21,30-tetrone	Detected (Harinantenaina Rakotondraibe et al., 2015)
Flavonoids	Mulberrin	Not investigated
	5-hydroxy-2-(4-hydroxyphenyl)-7-methoxy-4H-chromen-4-one	Anti-inflammatory (Yoo et al., 2025)
Polyketides/Complex secondary metabolites	(7S,9E,11S,12R,13S,14S,15S,16R,17S,18S,19E,21Z)-2,15,17-trihydroxy-11-methoxy-3,7,12,14,16,18,22-heptamethyl-26-(octylamino)-6,23,27,29-tetraoxo-8,30-dioxa-24-azatetracyclo[23.3.1.1^{4,7}0.0^{5,28}]triaconta-1,3,5(28),9,19,21,25-heptaen-13-yl acetate	Detected in lactic acid bacteria (Molina et al., 2025)
Terpenoid-derived aromatic esters	2-((((5aR,5bR,11aR)-9-((2-carboxybenzoyl)oxy)-5a,5b,8,8,11a-pentamethyl-1-(prop-1-en-2-yl)icosahydro-1H-cyclopenta[a]chrysen-3a-yl)methoxy)carbonyl)benzoic acid	Not investigated

* For compounds annotated as “not investigated” or “detected,” no direct biological activity has been experimentally demonstrated to date, and their inclusion reflects detection or structural characterization rather than confirmed functional activity.

## 4 Conclusion

Overall, UTNCys2-2 represents a genomically stable and biosafe strain with a compact, plasmid-free genome, enriched carbohydrate-active enzymatic capacity, and diverse biosynthetic potential, underscoring its relevance for EPS-driven functional fermentation, probiotic development, and microbial biotechnology applications. The presence of a functional Type II-A CRISPR-Cas system, balanced prophage content, and absence of virulence or transferable antimicrobial resistance genes underscore its genomic integrity and suitability for food and probiotic applications. Genomic evidence for EPS biosynthesis, glycogen metabolism, and wall teichoic acid production suggests roles in fermentation performance, stress tolerance, host interaction, and probiotic functionality. The complete riboflavin biosynthesis pathway further enhances its potential as a nutritionally beneficial starter or probiotic. A low-similarity T3PKS cluster indicates potential for novel secondary metabolite production, which may contribute to antimicrobial activity and ecological competitiveness. Pangenome analysis revealed high genomic plasticity and strain-specific genes associated with carbohydrate metabolism and cell surface structures, reflecting adaptability across ecological niches. Metabolomic profiling of the CFS demonstrated that antimicrobial and probiotic effects result from a synergistic network of alkaloids, bioactive peptides, functional carbohydrates, and phenolic compounds, supporting microbial inhibition, gut microbial balance, and host health. These findings establish *W. confusa* UTNCys2-2 as a biosafe, multifunctional strain with strong potential for applications in food fermentation, functional foods, and microbial biotechnology. Future studies should integrate transcriptomic and proteomic analyses to clarify regulation of carbohydrate metabolism, stress response, alongside *in vivo* assessments of adhesion, immunomodulation, and microbiota interactions, as well as pilot-scale fermentation trials to validate technological performance and safety in real food matrices.

## Data Availability

The genome assembly data of UTNCys2-2 have been deposited in the NCBI Sequence Read Archive under BioProject ID PRJNA1116628 (https://www.ncbi.nlm.nih.gov/sra/PRJNA1116628) and BioSample accession SAMN48695879.

## Footnotes

1
https://metacyc.org
