# Supplementary material for: Comprehensive genomic and metabolomic profiling of Weissella confusa UTNCys2-2 highlights bioactive potential
Source: Front Microbiol. 2026 Feb 27;17:1779198. doi: 10.3389/fmicb.2026.1779198 (PMC12982343; doi:10.3389/fmicb.2026.1779198)

**Supplementary files**

**Comprehensive Genomic and Functional Characterization of *Weissella confusa* isolated from Amazonian spiral-ginger**

Gabriela N. Tenea

Biofood and Nutraceutics Research and Development Group; Faculty of Engineering in Agricultural and Environmental Sciences, Universidad Técnica del Norte. Av. 17 de Julio s-21 y José María Córdova. 100105, Ibarra, Ecuador.

Corresponding author: gntenea@utn.edu.ec

**Table S1.** The best-hit BLASTN analysis

| ***Contig*** | **Contig Length** | **Subject Description** | **Subject Length** | ***E-Value*** |
| --- | --- | --- | --- | --- |
| contig 1 | 468,715 | *CP080582.1 Weissella confusa strain LM1* | *2,503,583* | 0.00 |
| contig 2 | 319,109 | *CP027563.1 Weissella confusa strain VTT* | *2,212,145* | 0.00 |
| contig 3 | 260,472 | *CP049097.1 Weissella confusa strain N17* | *2,279,677* | 0.00 |
| contig 4 | 205,025 | *CP080582.1 Weissella confusa strain LM1* | *2,503,583* | 0.00 |
| contig 5 | 183,437 | *CP080582.1 Weissella confusa strain LM1* | *2,503,583* | 0.00 |
| Contig : The name of contig. | | |  |  |
| Contig length (bp) : The total number of bases in the contig. | | |  |  |
| Subject description : Description of sequence matched by BLASTN. | | | |  |
| Subject length (bp) : Length of the sequence matched by BLASTN. | | | |  |
| E-value : The expectations that could be matched by chance. The lower: the more significant. | | | | |

**Table S2**. List of genomes used for pangenome analysis

| **Assembly** | **Strain** |
| --- | --- |
| [ASM477107v1](https://www.ncbi.nlm.nih.gov/datasets/genome/GCF_004771075.1/) | *Weissella confusa VTT E-133279.* |
| [ASM477129v1](https://www.ncbi.nlm.nih.gov/datasets/genome/GCF_004771295.1/) | *Weissella confusa VTT E-90392* |
| [ASM1957615v1](https://www.ncbi.nlm.nih.gov/datasets/genome/GCF_019576155.1/) | *Weissella confusa LM1* |
| [ASM2953620v1](https://www.ncbi.nlm.nih.gov/datasets/genome/GCF_029536205.1/) | *Weissella confusa CYLB30* |
| [ASM2596038v1](https://www.ncbi.nlm.nih.gov/datasets/genome/GCF_025960385.1/) | *Weissella confusa WiKim51* |
| [ASM1104433v1](https://www.ncbi.nlm.nih.gov/datasets/genome/GCF_011044335.1/) | *Weissella confusa N17* |
| [ASM1646661v1](https://www.ncbi.nlm.nih.gov/datasets/genome/GCF_016466615.1/) | *Weissella confusa LMG 17705* |
| [ASM1469454v1](https://www.ncbi.nlm.nih.gov/datasets/genome/GCF_014694545.1/) | *Weissella confusa UC4052* |
| [ASM195109v1](https://www.ncbi.nlm.nih.gov/datasets/genome/GCF_001951095.1/) | *Weissella cibaria CMS3* |
| [ASM991391v1](https://www.ncbi.nlm.nih.gov/datasets/genome/GCF_009913915.1/) | *Leuconostoc mesenteroides SRCM102733* |

**Table S3.** The top 5 genomes resulted by ANI analysis.

| **Ranking** | **Similar genome** | **ANI (%)** | **Aln. Cov.** |
| --- | --- | --- | --- |
| 1 | *W. confusa* DRR221367_bin.8_MetaWRAP_v1.3_MAG | 98.57 | 74.34 |
| 2 | *W. confusa* SRR11489763_bin.10_metaWRAP_v1.3_MAG | 98.54 | 54.01 |
| 3 | *W. confusa* strain 744 | 98.41 | 18.29 |
| 4 | *W. confusa* 1A-dyr2-07_110104.29_1677751023 | 98.36 | 40.18 |
| 5 | *W. confusa* NNI | 98.35 | 89.01 |

Ranking : The order of the highest ANI value.

 Similar genome : Species name which has high ANI value.

 ANI : % of relatedness at whole-genome level.

 Aln. Cov. : Alignment coverage. % of coverage by assembly sequence alignments against compared genome. ANI: Average Nucleotide Identity

**Figure S1.** Genomic landscape of UTNCys2-2 with a complete Type II-A CRISPR-Cas system, intact prophages, and antimicrobial resistance genes, emphasizing its defense capacity and genomic versatility.

**
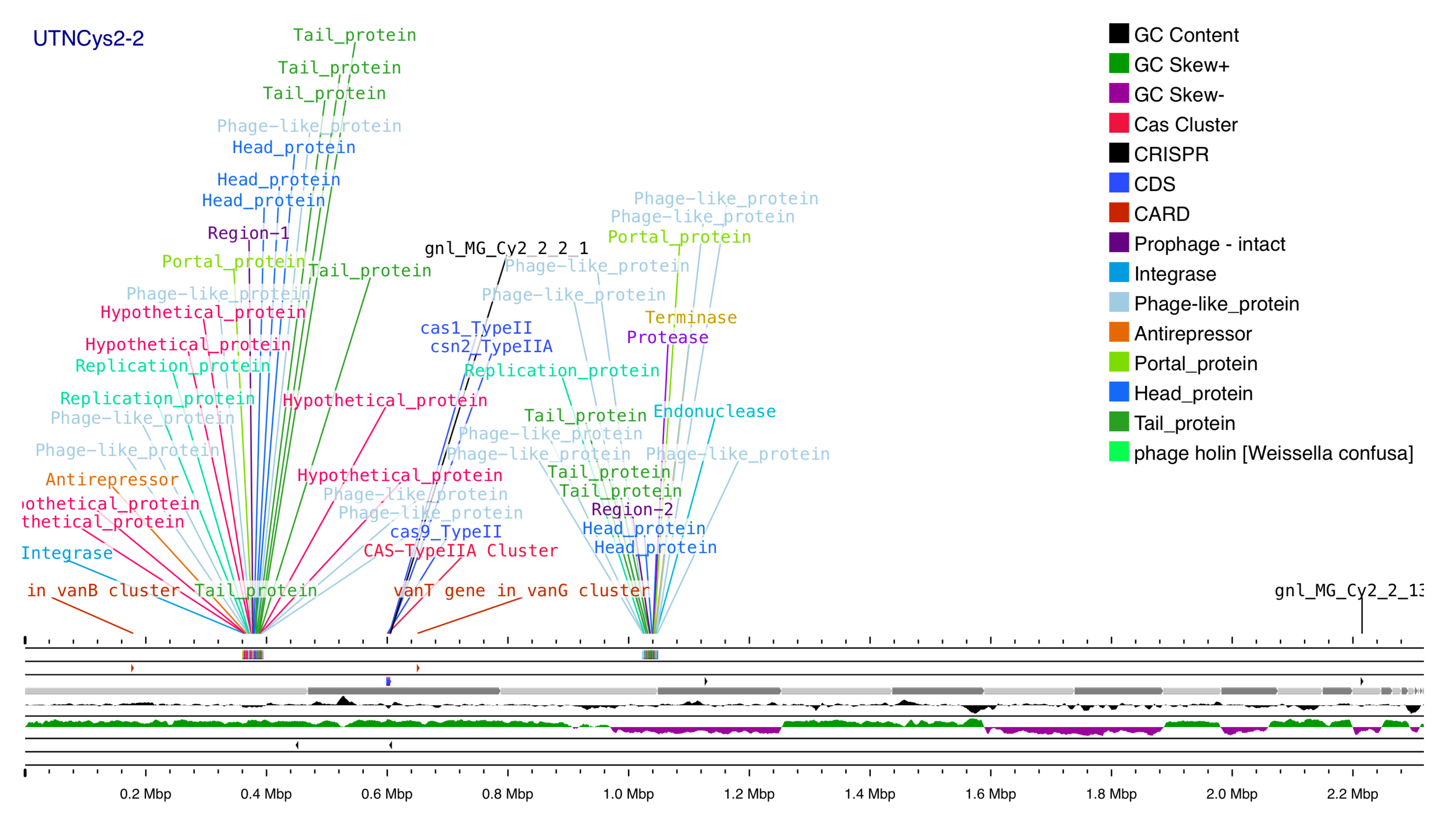
**

**Figure S2.** KEGG pathway map of starch and sucrose metabolism. Enzymes highlighted in **blue** indicate genes significantly expressed or involved in the EPS biosynthetic pathway. This pathway includes various intermediates such as sucrose, glucose, and fructose derivatives, which are precursors for EPS production. The integration with glycolysis and amino sugar metabolism (as indicated by dashed arrows) supports carbon flux toward polysaccharide synthesis. (Adapted from KEGG Pathway Database)

**
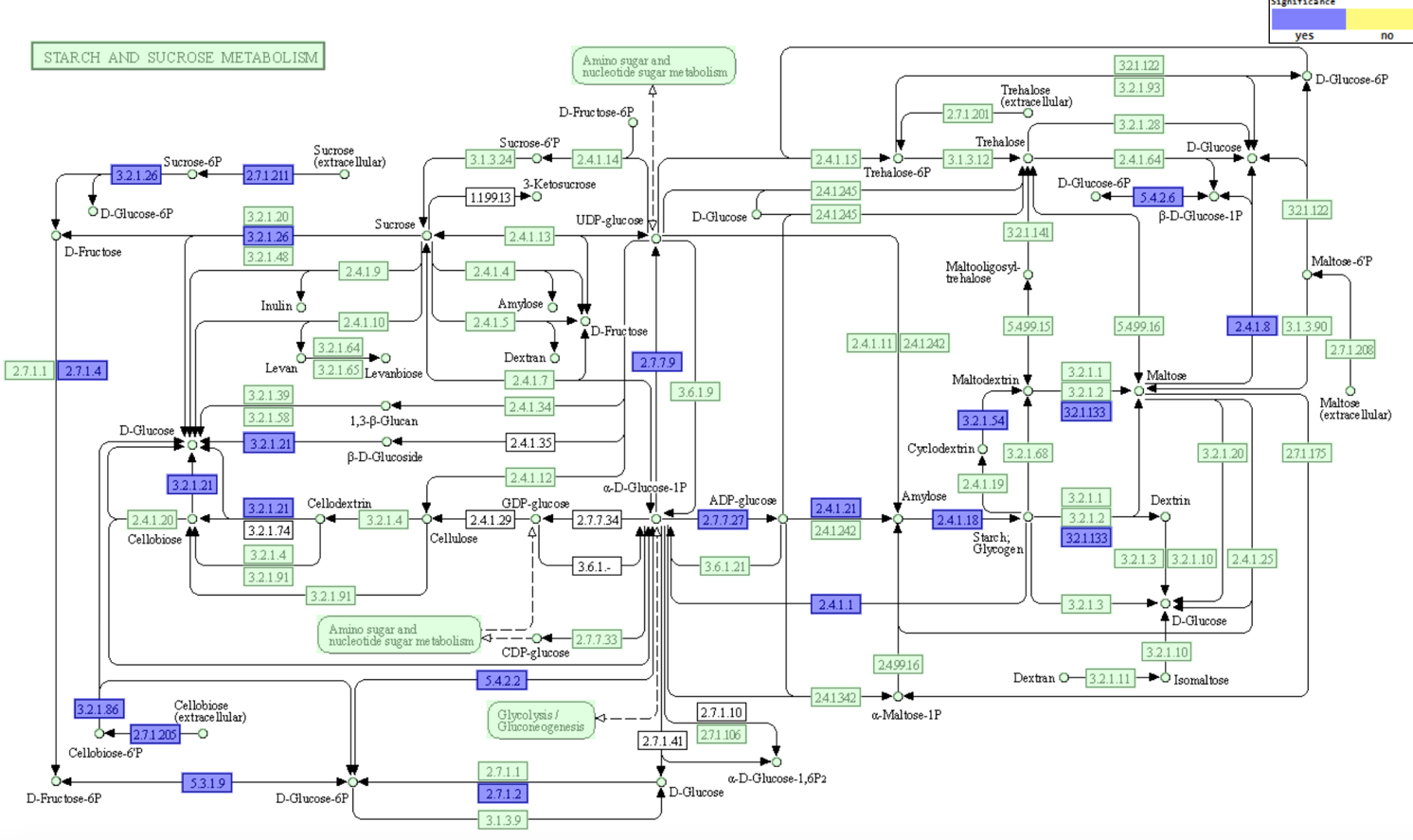
**

**Figure S3.** KEGG pathway map of amino sugar and nucleotide sugar metabolism. Enzymes highlighted in **blue** represent genes significantly expressed or associated with exopolysaccharide (EPS) biosynthesis. This pathway contributes essential sugar nucleotides like UDP-glucose, UDP-galactose, and UDP-N-acetylglucosamine, which serve as direct precursors for EPS formation. Dashed arrows indicate cross-pathway links to peptidoglycan biosynthesis and glycolysis. (Adapted from KEGG Pathway Database)


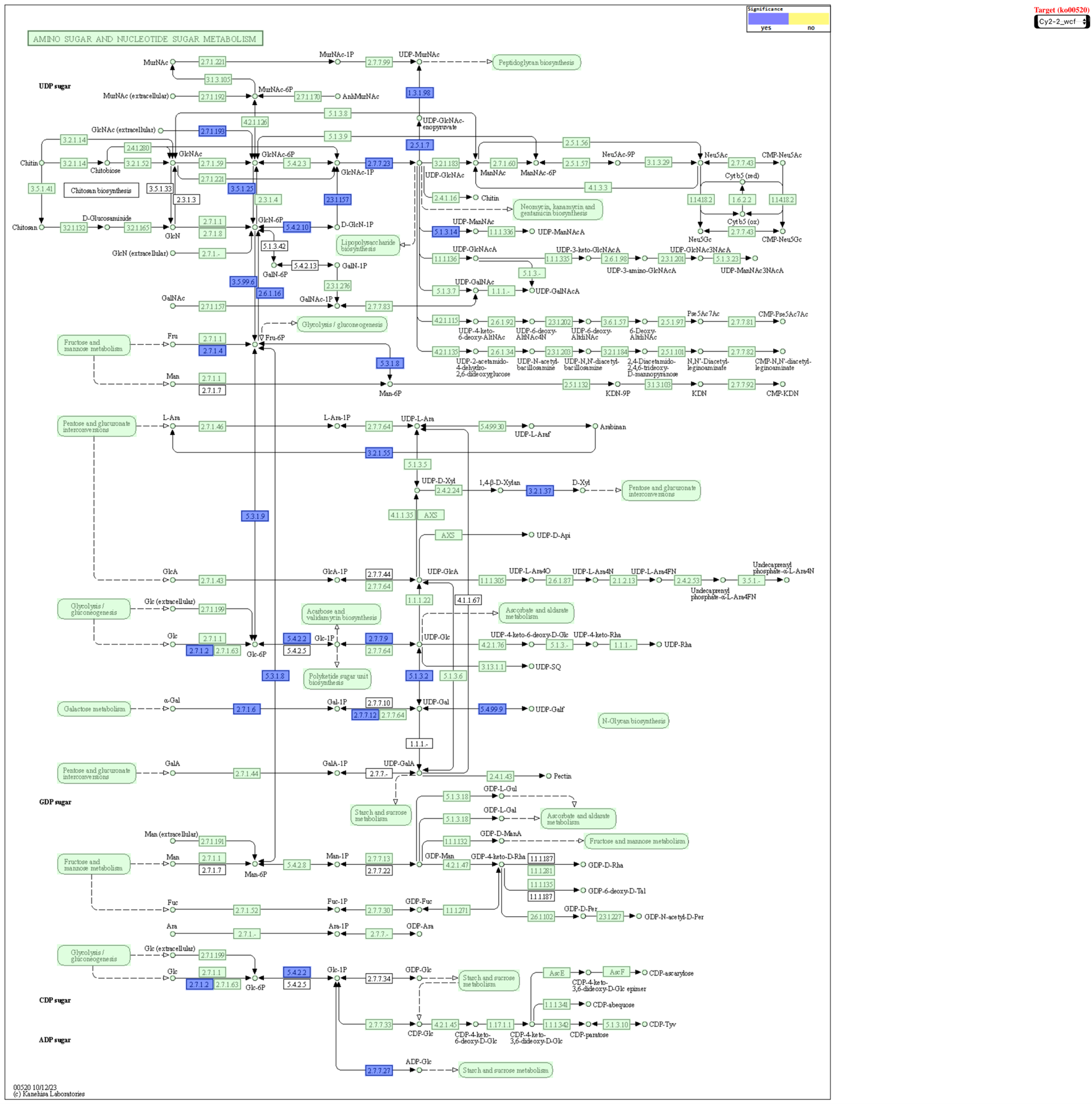


**Figure S4**. Pangenome Roary matrix. Gene content comparison of the 10 considered strains. The matrix shows genes typical of each strain and those conserved in all.


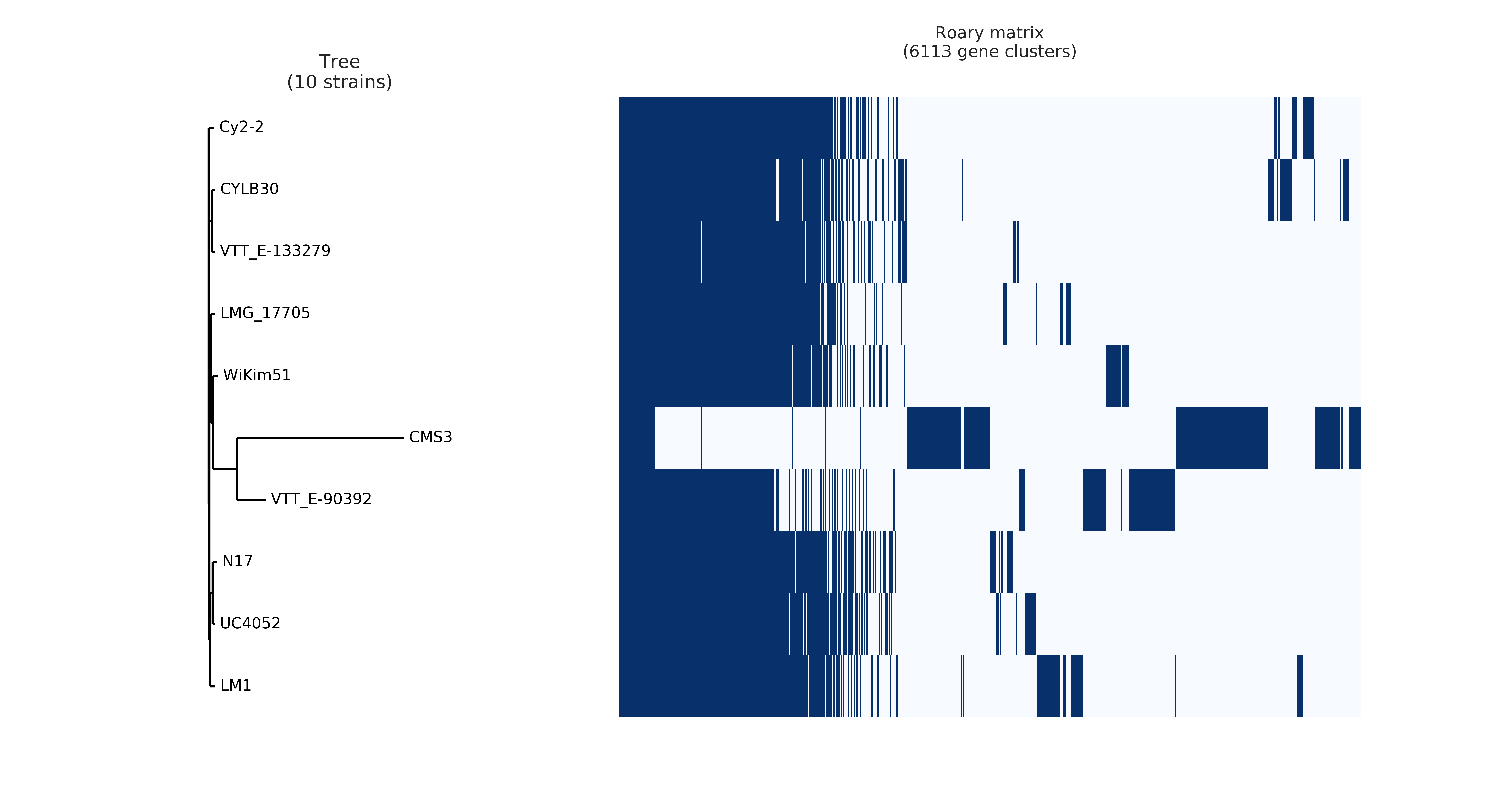

Supplement: Supplementary file 1 [file Data_Sheet_1.docx]
